# Description and management of patients with anal fissure: insights on Italian primary care setting coming from real-world data

**DOI:** 10.1007/s13304-024-01882-8

**Published:** 2024-05-26

**Authors:** Gaetano Gallo, Valeria Pegoraro, Mario Trompetto

**Affiliations:** 1 Department of Surgery, Sapienza University of Rome, Viale Regina Elena, 324, 00161 Rome, RM Italy; 2 IQVIA Solutions Italy Srl, Milan, Italy; 3 Department of Colorectal Surgery, S. Rita Clinic, Vercelli, Italy

**Keywords:** Anal fissure, Real-world evidence, Retrospective, Primary care, General practitioners, Topical nifedipine and lidocaine combination

## Abstract

To describe patients with anal fissure (AF) and their management in primary care. Retrospective study using the Italian Longitudinal Patient Database on 18 + years old subjects with AF records during ‘July 2016–June 2021’ (selection period). Index Date (ID) was the first AF record during selection period. Sub-cohorts were defined by presence/absence of prescriptions on ID of the combination of topical nifedipine 0.3% and lidocaine 1.5% (NIF/LID). Patients’ information on the 12-month period before (baseline) and after (follow-up) ID was analyzed. Subjects with AF were 8632: 14.0% had NIF/LID on ID. Mean age was 52 (± 17.2) years, there were more women in ‘ < 50 years’ group, and more men in ‘50–70’ one. Prevalences of pregnancy and immunodepression were around 5%; most common comorbidities were hypertension (29.6%) and heart disease (13.1%), while constipation and diarrhea were < 5%. Healthcare resources utilization (HRUs) increased during follow-up, but still few patients were prescribed NIF/LID (2.8%), other treatments for AF (10.3%), or proctological visits (7.7%). NIF/LID patients were younger (< 40 years people: 30.7% versus 23.9%; *p* value < 0.0001), and more likely to have constipation (4.3% versus 2.5%; *p* value < 0.001); patients without NIF/LID showed slightly higher prevalences of hypertension (30.0% versus 27.1%; *p* value: 0.039) and depression (4.0% versus**2.5%; *p* value: 0.009), and a little higher overall HRUs. Results show that general practitioners are used to manage AF. However, there is still a gap between guidelines’ recommendations and actual management. Educational campaigns on common anal problems in primary care might help further improving AF management and optimizing HRUs.

**Supplementary Information:**

The online version contains supplementary material available at 10.1007/s13304-024-01882-8.

## Introduction

Anal fissure (AF), one of the common pathologies of the anal canal which is associated with a greater anal sphincter pressure [1, 2], is a longitudinal tear within the anal canal that usually extends from the dentate line toward the anal verge [3]. The etiology of AF is not clear [4, 5]. A common trigger is the trauma due to passing a large or hard stool, but many traumatic fissures heal and others do not [6]. AFs can also be associated with complex multisystem conditions like Crohn’s disease and human immunodeficiency virus (HIV) [7]. AFs can be classified as acute or chronic depending on time of onset and morphology. In particular, AFs are considered acute when they are of recent onset (less than 6 weeks), and chronic when they are present for a longer period [8]. Acute AFs typically present as linear lesions with clear margins; chronic AFs are wider and deeper and present with granulation tissue at the base and potential exposure of the internal sphincter [8] taking on a characteristic aspect that includes perianal skin tag, fibrotic edges, and a proximal papilla [9].

Few data are available on AFs epidemiology: a population-based study conducted in 2014 by Mapel and colleagues in the United States found an overall incidence of AF of 1.1 per 1000 person-years, which translated into an average lifetime risk of 7.8% [10]. Indeed, AF is a common condition that causes significant complications in a young and otherwise healthy population [11], as peak incidence occurs between 15 and 40 years of age [7]. AF usually causes pain during defecation, which may last for 1–2 h [6] and it is usually sharp, causing a tearing sensation, and it is debilitating because of the intensity. AFs are the second most common cause after hemorrhoids for proctology visits in Italy [12].

Management options of AF include both primary and secondary care interventions [6, 7], all aimed to reduce the anal tone [6]. According to American Society of Colon and Rectal Surgeons (ASCRS) guidelines, non-operative treatment of acute AFs is safe and should be first-line treatment [3]. Indeed, about half of the patients with acute AF has shown to respond to non-operative measures, and topical calcium channel blockers (CCBs) should be regarded as first-line options [3].

To authors’ knowledge, no data are available on the management of AF by general practitioners (GPs) in Italy. For this reason, the present study used real-world data from a large sample of Italian GPs with the main objective of describing patients receiving a diagnosis of AF in a primary care context in terms of demographic and clinical characteristics, and healthcare resources utilization (HRUs).

## Methods

### Data source

This was a real-world retrospective study using data from the IQVIA Italian Longitudinal Patient Database (LPD). IQVIA LPD provides insights from ~ 900 GPs providing data of approximately 1.2 million patients, who are representative of the Italian general population managed by GPs in terms of age and gender. Representativeness of IQVIA LPD has been previously demonstrated by comparing patients’ distributions of age and sex with the Italian National Institute of Statistics [13]. According to Italian law requirements, collaborating GPs use an ambulatory management software to record information on their patients’ routine visits. GPs voluntarily agreed to contribute to the database and attended specific trainings for data entry. Indeed, GPs shall meet up-to-standard quality criteria related to the levels of coding, prevalence of well-known diseases, mortality rates, and years of recording to be considered for participation in epidemiological studies. Recorded data include demographic information of the patients, diagnoses, drug prescriptions, and referrals for specialist visits, and examinations. The codification system of diagnoses follows the International Classification of Diseases 9th revision (ICD-9), while that of drugs complies with the Anatomical Therapeutic and Chemical (ATC) classification. Italian IQVIA LPD has been shown to be a reliable source of information in numerous previous studies and disease areas [14–19] and has been recently added to the European Medicine Agency (EMA) Real-World Data Catalogue [20]. IQVIA LPD relies on anonymous data which are not originated by any clinical trial. A non-identifiable encryption process guarantees data anonymization directly on GPs’ computer, thus before storage into IQVIA databases. IQVIA LPD complies with the European Regulation 679/2016 and the ex-Legislative Decree 196/03 and subsequent modifications, and all the analyses using IQVIA LPD data do not require any Ethical Committee’s approval.

### Study populations and study design

**Fig. 1:**
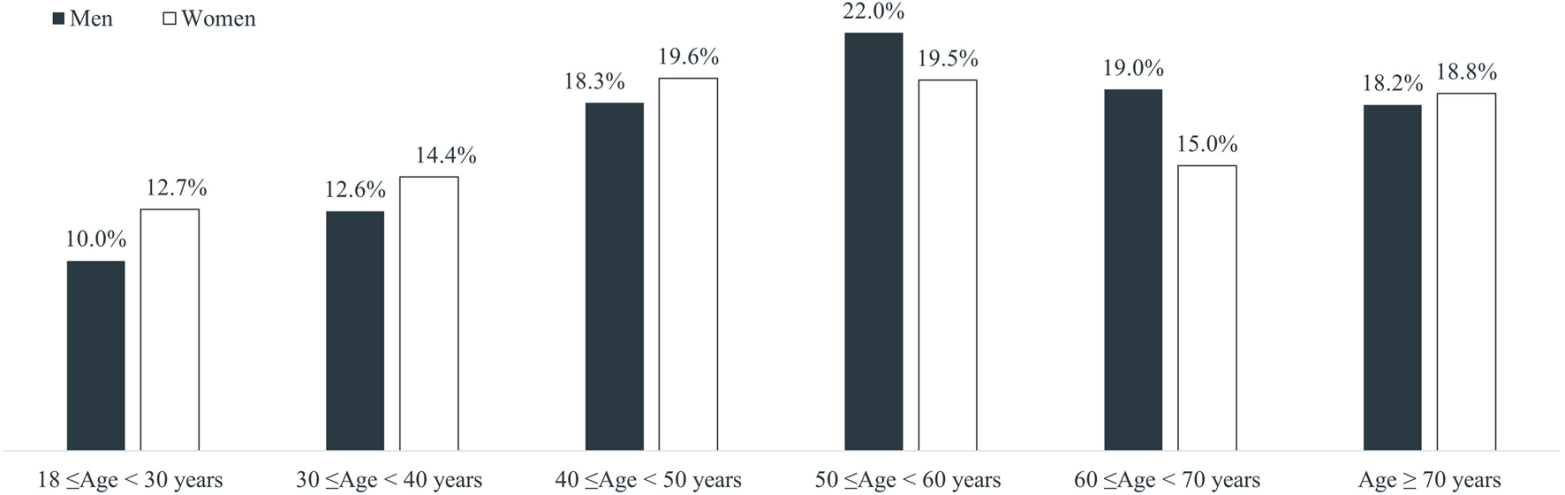
Patients with anal fissure (AF) stratified by age class and sex. Analysis on the overall cohort

We first included subjects aged 18 years or older with at least one occurrence of a record of a health encounter related to a diagnosis of AF (ICD-9 code 565.0) during the period 1st July 2016–30th June 2021 (i.e., selection period). An Index Date was defined for each subject according to the date of registration of the first AF record during the selection period. Records of health encounters related to AF could preexist. To be included in the study, a patient had to have data availability for the entire study period, i.e., the 12-month period preceding the Index Date (i.e., baseline period) and the 12-month period starting at the Index Date (i.e., follow-up period). All subjects meeting the above criteria recorded in the database were considered for this study. Indeed, the more data that are available, the higher the level of confidence for any estimate provided. In particular, a sample size of at least 384 subjects is the largest sample size required to determine a proportion with a precision of ± 5% with a two-sided Type I error of 0.05 according to the sample size formula based on the normal approximation to the binomial distribution. Furthermore, a preliminary assessment was performed and all drugs’ prescriptions for which the GP recorded a specific diagnosis of AF as underneath cause found during the period 1st July 2015–30th June 2022 were extracted from the database. Ten most frequently prescribed molecules were provided, and prescriptions of the combination of topical nifedipine 0.3% and lidocaine 1.5% (NIF/LID), which is a treatment for AFs and proctalgia typically associated with anal sphincter hypertonia [21], accounted for approximately one-fifth of total prescriptions (please see Supplementary Material,Fig. 1S). Because (1) the second most frequently prescribed treatment was mesalazine (14%), which is not specifically indicated for the treatment of AF, but it is an anti-inflammatory drug used to treat inflammations of the digestive tract such as ulcerative colitis and mild-to-moderate Crohn's disease [22], (2) other molecules accounted for no more than 5% of prescriptions, subjects included in the final cohort were then grouped into two different sub-cohorts depending on presence/absence of NIF/LID prescription at Index Date to perform an exploratory investigation on prescription habits of Italian GPs toward AF.

### Information extracted from the database

Information extracted from the database to characterize study patients included subjects’ age and sex at Index Date, conditions and comorbidities of interest during baseline, drugs’ prescriptions related to AF, proctological visit and diagnostic examinations referrals both during baseline and follow-up. Conditions of interest investigated were pregnancy, immunosuppressive condition, and assumption of anti-thrombotics (ATC code B01A). Please see Supplementary Material, Tables 1S and 2S, for the list of ICD-9 codes, referrals, and ATC codes used to define conditions of interest. Comorbidities of interest included constipation, diarrhea, diabetes mellitus, hypertension, hypothyroidism, obesity, anxiety, depression, chronic inflammatory bowel diseases, and heart diseases. Please see Supplementary Material, Table 3S, for the list of ICD-9 codes used to define comorbidities. Drugs prescriptions related to AF included those of agents for treatment of hemorrhoids and anal fissures for topical use (i.e., ATC code C05A: corticosteroids, antibiotics, local anesthetic, muscle relaxants, and other agents for treatment of hemorrhoids and anal fissures for topical use), cicatrizants (ATC code D03A), and those of analgesics (ATC code N02, excluding antimigraine preparations) when specifically prescribed in relation with a diagnosis for AF. Finally, referrals were extracted from the database where those requesting a proctological visits or a diagnostic examination of interest. Diagnostic examinations considered were colonoscopy, anoscopy, rectoscopy, anorectal manometry, sigmoidoscopy, defecography, transrectal ultrasounds, abdomen or pelvic magnetic resonance, abdomen or pelvic ultrasounds.

### Statistical analysis

Patients’ demographic and clinical characteristics, and HRUs were summarized overall to respond to the main objective of the study, and also across sub-cohorts defined by the presence/absence of NIF/LID prescriptions at Index Date. Differences between sub-cohorts were examined using Chi-square statistics, and Yates correction was applied in case of counts lower than 5. *P* values <0.05 were considered statistically significant. To compare HRUs between numerically balanced sub-cohorts and to mitigate bias that might have been introduced by patients’ demographic characteristics, a sensitivity analysis was planned. In particular, a greedy nearest neighbor with a specified caliper distance of 0.20 propensity score matching (PSM) was applied to perform a without replacement 1:1 matching of patients with and without NIF/LID prescriptions at Index Date. Covariates included in the propensity score model were patients’ age and sex. Goodness of the procedure was assessed using standardized mean differences, which were required to be lower than 0.1, which is the recommended upper limit [23], for all covariates. All the analyses were performed using SAS Enterprise Guide 8.2.

## Results

Patients with at least one record related to a diagnosis of AF during the selection period were 9343. After having excluded 153 (1.6%) subjects who were <18 years old and 558 (6.0%) who did not have data availability for the entire study period, the final cohort was composed of 8632 patients with AF. The preliminary assessment on prescriptions related to a diagnosis of AF found NIF/LID to be the most frequently prescribed molecule (17.9% of all prescriptions), followed by mesalazine (14.1%); beclomethasone, diosmin combinations, hydrocortisone, amoxicillin and enzyme inhibitors, macrogol combinations, combination of argentic sulfadiazine and hyaluronic acid, ketorolac, and combinations of codeine and paracetamol accounted for no more than 5% of all prescriptions each (Fig. 1S). Being so, patients with AF were further grouped into two different sub-cohorts: patients with NIF/LID prescription at Index Date (1209; 14.0%) and patients without NIF/LID prescription at Index Date (7423; 86.0%). On average, subjects with AF were 52 years old [mean ± SD: 52.4 ± 17.2; median (1st quartile; 3rd quartile): 52.0 (40.0; 66.0)], and men and women were equally distributed within the overall cohort (Table 1). However, the joint distribution of age and sex showed a higher prevalence of female gender among the <50 years old age group (46.7% versus 40.9%), and a higher prevalence of men in the population aged between 50 and 70 years old (41.0% versus 34.5%) (Fig. 1).

**Table 1:** Demographic and baseline clinical characteristics of patients with anal fissure (AF)

Patients’ characteristics	Total (*N* = 8632)		NIF/LID yes (*N* = 1209)		NIF/LID no (*N* = 7423)		*P* value^*1*^
Sex: n (%)							
Male	4406	(51.0)	618	(51.1)	3788	(51.0)	0.956
Female	4226	(49.0)	591	(48.9)	3635	(49.0)	
Age class at Index Date: n (%)							
18–29	979	(11.3)	201	(16.6)	778	(10.5)	< *0.0001*
30–39	1166	(13.5)	171	(14.1)	995	(13.4)	
40–49	1631	(18.9)	229	(18.9)	1402	(18.9)	
50–59	1793	(20.8)	232	(19.2)	1561	(21.0)	
60–69	1467	(17.0)	185	(15.3)	1282	(17.3)	
70 +	1596	(18.5)	191	(15.8)	1405	(18.9)	
Conditions of interest^2^							
Pregnancy^3^	218	(5.2)	38	(6.4)	180	(5.0)	0.132
Immunosuppression	423	(4.9)	54	(4.5)	369	(5.0)	0.451
Assumption of antithrombotic agents	1625	(18.8)	225	(18.6)	1400	(18.9)	0.837
Comorbidities of interest^2^							
Constipation	238	(2.8)	52	(4.3)	186	(2.5)	< *0.001*
Diarrhea	265	(3.1)	39	(3.2)	226	(3.0)	0.735
Diabetes mellitus	644	(7.5)	90	(7.4)	554	(7.5)	0.981
Essential hypertension	2552	(29.6)	327	(27.1)	2225	(30.0)	*0.039*
Hypothyroidism	479	(5.6)	56	(4.6)	423	(5.7)	0.133
Obesity	593	(6.9)	81	(6.7)	512	(6.9)	0.801
Anxiety	457	(5.3)	59	(4.9)	398	(5.4)	0.488
Depression	330	(3.8)	30	(2.5)	300	(4.0)	*0.009*
Chronic inflammatory bowel disease	93	(1.1)	14	(1.2)	79	(1.1)	0.770
Heart disease	1127	(13.1)	147	(12.2)	980	(13.2)	0.318
Number of comorbidities of interest							
0	4461	(51.7)	681	(56.3)	3780	(50.9)	< *0.001*
1	2355	(27.3)	280	(23.2)	2075	(28.0)	
2 +	1816	(21.0)	248	(20.5)	1568	(21.1)	

Analysis on the overall cohort and on sub-cohorts defined by presence/absence of nifedipine 0.3% in combination with lidocaine 1.5% (NIF/LID) prescription at Index Date. NIF/LID: nifedipine 0.3% in combination with lidocaine 1.5%

^1^*P* values < 0.05 were considered statistically significant

^2^Numbers and proportions of patients with at least one recording of the corresponding condition/diagnosis. One patient can be counted in more than one group

^3^Proportions calculated over the total number of women

Both pregnant women and immunodepressed subjects accounted for around 5% of the overall cohort, while patients prescribed anti-thrombotics represented about one-fifth (Table 1). Most frequently reported comorbidities were essential hypertension (29.6%) and heart disease (13.1%), followed by diabetes mellitus (7.5%), obesity (6.9%), hypothyroidism (5.6%), and anxiety (5.3%). Constipation, diarrhea, depression, and chronic inflammatory bowel disease did not reach 5% of the total cohort (Table 1). While sex distribution did not vary according to the presence/absence of NIF/LID prescription at Index Date, there was a statistically significant difference between the two sub-cohorts in terms of age distribution, with patients receiving NIF/LID at Index Date being younger. Indeed, 40 years younger people accounted for 30.7% of the sub-cohort with NIF/LID prescription versus 23.9% of the sub-cohort without NIF/LID prescription at Index Date; on the other hand, 60+ years old patients represented 31.1% and 36.2% of the sub-cohorts with and without NIF/LID prescription at Index Date, respectively (Table 1). No relevant differences were observed either in terms of conditions, or in terms of comorbidities of interest, with just slightly higher proportions of patients with constipation (4.3 versus 2.5%) among patients with NIF/LID prescription at Index Date, and slightly higher proportions of patients with essential hypertension (30.0 versus 27.1%) and depression (4.0 versus 2.5%) among subjects without NIF/LID prescription at Index Date. Finally, a higher proportion of patients without any comorbidity of interest was found in the sub-cohort with NIF/LID prescription at Index Date (56.3 versus 50.9%) (Table 1).

HRUs was higher during follow-up, than during baseline. Overall, the proportion of patients with at least one prescription for treatments of interest increased from 3.0 to 10.3% (+243%); subjects with at least one referral for a proctological visit accounted for 3.9% during baseline and for 7.7% during follow-up (+97%); people with at least one referral for a diagnostic examination represented 17.2 and 21.5% of the total cohort during baseline and follow-up, respectively (+25%) (Table 2). The same trend of increasing HRUs between baseline and follow-up was observed when looking at the two sub-cohorts. Slightly but significantly higher proportions of patients with referrals for proctological visits and diagnostic examinations during baseline were found within the sub-cohort without NIF/LID prescription at Index Date; all the HRUs items considered (treatments, proctological visits, diagnostic examination) were more frequently reported during follow-up for patients without NIF/LID prescription at Index Date, even if significant difference was reached only for diagnostic examinations (21.9 versus 18.6%). While the magnitude of HRUs increase between baseline and follow-up was higher among subject without NIF/LID prescription at Index Date for treatments of interest (+250% versus 174%) and diagnostic examinations (25% versus 23%), the opposite occurred for proctological visits, with an increase of 97% among subjects with NIF/LID prescription at Index Date versus 93% among subjects without NIF/LID prescription at Index Date. Finally, proportions of patients receiving NIF/LID after the Index Date were significantly higher among those who already received NIF/LID at Index Date (7.0 versus 2.1%).

**Table 2:** Baseline and follow-up healthcare resources utilization (HRUs) of patients with anal fissure (AF)

HRUs	Total (*N* = 8632)		NIF/LID yes (*N* = 1209)		NIF/LID no (*N* = 7423)		P value^1^
Baseline							
Treatments of interest^2^							
At least one prescription	260	(3.0)	41	(3.4)	219	(3.0)	0.406
Proctological visit							
At least one referral	337	(3.9)	34	(2.8)	303	(4.1)	*0.035*
Diagnostic examination^3^							
At least one referral	1484	(17.2)	182	(15.1)	1,302	(17.5)	*0.034*
Follow-up							
Treatments of interest^2^							
At least one prescription	889	(10.3)	113	(9.3)	776	(10.5)	0.240
Proctological visit							
At least one referral	666	(7.7)	83	(6.9)	583	(7.9)	0.232
Diagnostic examination^3^							
At least one referral	1852	(21.5)	225	(18.6)	1,627	(21.9)	*0.009*
NIF/LID^4^							
At least one prescription	243	(2.8)	85	(7.0)	158	(2.1)	< *0.0001*

Analysis on the overall cohort and on sub-cohorts defined by presence/absence of nifedipine 0.3% in combination with lidocaine 1.5% (NIF/LID) prescription at Index Date. NIF/LID: nifedipine 0.3% in combination with lidocaine 1.5%. ATC: anatomical therapeutic chemical classification. AF: anal fissure. HRUs: healthcare resources utilization

^1^*P* values < 0.05 were considered statistically significant

^2^Treatments considered included agents for treatment of hemorrhoids and anal fissures for topical use (ATC class C05A) (excluding NIF/LID), analgesics (ATC class N02) (when prescribed for a diagnosis of AF and excluding antimigraine preparations), and cicatrizants (ATC class D03A)

^3^Diagnostic examinations considered included colonoscopy, anoscopy, rectoscopy, sigmoidoscopy, anorectal manometry, defecography, transrectal ultrasounds, abdominal ultrasounds, pelvic ultrasounds, pelvic magnetic resonance, abdominal magnetic resonance

^4^At least one prescription during follow-up, excluding Index Date

**Fig. 2:**
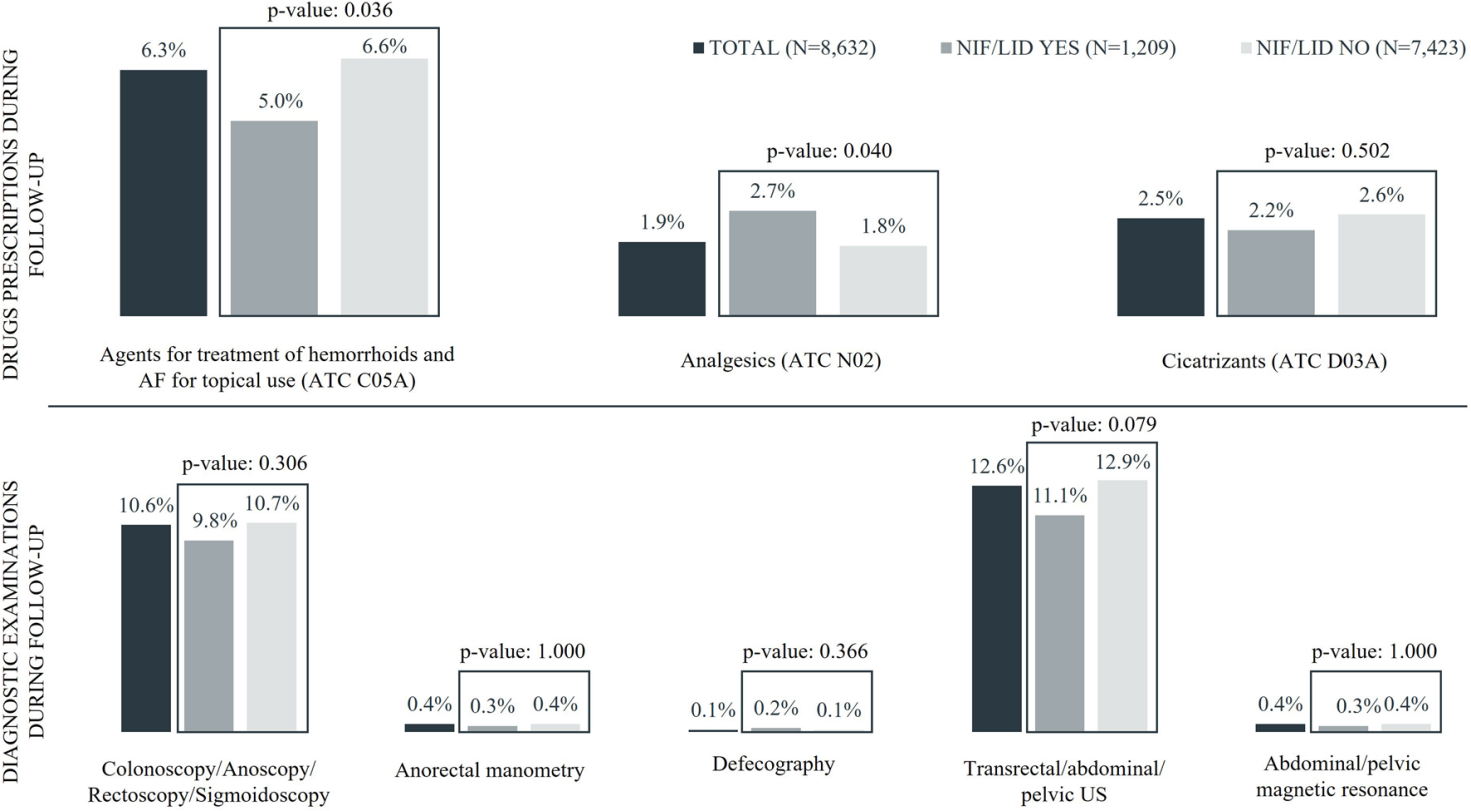
Details on follow-up drugs prescriptions and diagnostic examinations. Analysis on the overall cohort and on sub-cohorts defined by presence/absence of nifedipine 0.3% in combination with lidocaine 1.5% (NIF/LID) prescription at Index Date. NIF/LID: nifedipine 0.3% in combination with lidocaine 1.5%. AF: anal fissure. ATC: anatomical therapeutic chemical classification

Drugs falling into C05A ATC class (agents for treatment of hemorrhoids and AF for topical use) other than NIF/LID were the most frequently prescribed AF-related treatments during follow-up both overall and on the two sub-cohorts. The proportion of subjects receiving a C05A drug was significantly higher for the sub-cohort of patients without NIF/LID prescription at Index Date (6.6 versus 5.0%). Cicatrizants were prescribed to 2.5% of patients in the overall cohort with small differences between sub-cohorts, while analgesics, which were recorded for 1.9% of the overall cohort, were more frequently observed in the sub-cohort of subjects with NIF/LID prescription (2.7 versus 1.8%; *P* value: 0.04) (Fig. 2). Focusing on diagnostic examinations, proportions of patients with referrals for anorectal manometry, defecography, and abdominal/pelvic magnetic resonance were negligible, while 12.6% and 10.6% of patients in the overall cohort had referrals for transrectal/abdominal/pelvic ultrasound and for colonoscopy/anoscopy/rectoscopy/sigmoidoscopy, respectively; both types of examinations were more frequently observed among patients without NIF/LID prescription at Index Date, but differences were not statistically significant (Fig. 2).

To perform the sensitivity analysis on HRUs, two subgroups of matched patients were identified through PSM, each one composed of 1209 subjects, i.e., those with or without NIF/LID prescription at Index Date. Standardized mean differences resulting from PSM were <0.0005 for both sex and age, indicating goodness of matching. Indeed, sex and age distributions were identical, with proportions of males of 51.1% and an average age of about 50 years [mean ± SD: 49.9 ± 17.7; median (1st quartile; 3rd quartile): 50.0 (35.0; 63.0)] for both subgroups. Results from the sensitivity analysis on HRUs mainly confirmed those from the main analysis, even if the only statistically significant difference between sub-cohorts remained those in the proportion of patients receiving at least one NIF/LID after Index Date, which was higher among subjects with NIF/LID prescription at Index Date (Table 3), and in the proportion of patients with at least one C05A prescription during follow-up, which was higher among subjects without NIF/LID prescription at Index Date (Fig. 2S, Supplementary Materials).

**Table 3:** Baseline and follow-up healthcare resources utilization (HRUs) of patients with anal fissure (AF)

HRUs	Total (N = 2,418)		NIF/LID yes (N = 1,209)		NIF/LID no (N = 1,209)		P value^1^
Baseline							
Treatments of interest^2^							
At least one prescription	78	(3.2)	41	(3.4)	37	(3.1)	0.645
Proctological visit							
At least one referral	78	(3.2)	34	(2.8)	44	(3.6)	0.250
Diagnostic examination^3^							
At least one referral	366	(15.1)	182	(15.1)	184	(15.2)	0.910
Follow-up							
Treatments of interest^2^							
At least one prescription	251	(10.4)	113	(9.3)	138	(11.4)	0.096
Proctological visit							
At least one referral	186	(7.7)	83	(6.9)	103	(8.5)	0.127
Diagnostic examination^3^							
At least one referral	482	(19.9)	225	(18.6)	257	(21.3)	0.103
NIF/LID^4^							
At least one prescription	108	(4.5)	85	(7.0)	23	(1.9)	< *0.0001*

Sensitivity analysis on the overall cohort and on sub-cohorts defined by presence/absence of nifedipine 0.3% in combination with lidocaine 1.5% (NIF/LID) prescriptions at Index Date and 1:1 matched based on demographic characteristics. NIF/LID: nifedipine 0.3% in combination with lidocaine 1.5%. ATC: anatomical therapeutic chemical classification. AF: anal fissure. HRUs: healthcare resources utilization

^1^*P* values < 0.05 were considered statistically significant

^2^Treatments considered included agents for treatment of hemorrhoids and anal fissures for topical use (ATC class C05A) (excluding NIF/LID), analgesics (ATC class N02) (when prescribed for a diagnosis of AF and excluding antimigraine preparations), and cicatrizants (ATC class D03A)

^3^Diagnostic examinations considered included colonoscopy, anoscopy, rectoscopy, sigmoidoscopy, anorectal manometry, defecography, transrectal ultrasounds, abdominal ultrasounds, pelvic ultrasounds, pelvic magnetic resonance, abdominal magnetic resonance

^4^At least one prescription during follow-up, excluding Index Date

## Discussion

Main objective of the present study was to characterize patients receiving a diagnosis of AF in a primary care context in terms of demographic and clinical characteristics, and HRUs.

Results from our study showed that, on average, patients with AF were 52 years old, while sex distribution varies with age, with a higher prevalence of female gender among 50 years younger people, and a higher prevalence of men in the age group 50–70 years old. These findings are in line with those by Mapel and colleagues who reported relevant differences in AF incidence between men and women according to age [10]. While no differences were observed in terms of sex between the two sub-cohorts, patients prescribed with NIF/LID at Index Date were significantly younger than subjects without NIF/LID prescription at Index Date. Indeed, according to authors’ knowledge, NIF/LID is more often prescribed to patients with a major anal tone, which is known to decrease with increasing age [24]. Evidence of pregnancy was detected in 5% of women with AF, with this proportion doubling when considering women <50 years old, while immunodepressive condition was recorded in 5% of the overall cohort. The association between pregnancy condition and disease of the anal canal is known, with hemorrhoids and AFs occurring in about 40% of pregnant women and women after delivery [25]. Similarly, AFs have already been associated with complex multisystem conditions like Crohn’s disease and human immunodeficiency virus [7]. Among comorbidities of interest, the most frequently reported were hypertension, heart disease, diabetes mellitus, obesity, and hypothyroidism. Evidence of constipation and diarrhea, which have been shown to increase trauma and irritation of the anal canal potentially leading to development of AFs [7], were both reported in only around 3% of the patients. However, it is authors’ opinion that such symptoms might be underreported by GPs. No relevant differences were observed between sub-cohorts in terms of clinical characteristics, except higher proportions of subjects with constipation among patients prescribed with NIF/LID at Index Date, and higher prevalence of hypertension and depression among subjects without NIF/LID prescription at Index Date. An increase in HRUs was observed during follow-up, which is more evident when focusing on prescriptions of drugs and referrals for proctological visits. This seems to reveal an intention to manage AFs by Italian GPs; however, it should be mentioned that, overall and despite the observed increase, proportions of patients with drugs prescriptions related to AF and referrals for proctological visits were still quite low during follow-up, with this suggesting a potential room for improvement in the management of AFs. Proportions of patients with referrals for diagnostic examinations were higher than those observed for AF drugs and proctological visits. This might suggest that GPs who are used to seek for AF diagnosis confirmation more frequently rely on diagnostic examinations rather than turning to a proctologist. No relevant differences were observed between sub-cohorts in terms of HRUs, with just a slightly higher trend of utilization among patients without NIF/LID at Index Date. Results from the present study seems to reveal that evidence on the management of AF in primary care is somewhat controversial. Indeed, a lot of management options of AF do exist, including both primary care interventions, like lifestyle advice (e.g., dietary fiber supplements and adequate fluid intake), laxatives, muscle relaxants including topical treatments (e.g., glyceryl trinitrate (GTN) ointment, CCBs, and secondary care interventions, like botulinum toxin injections, and surgery (i.e., lateral sphincterotomy, advancement flap procedures, and fissurectomy) [6, 7]. A recent international survey which aimed to investigate on surgeons’ practice and preferences for AF treatment worldwide showed that the first treatment of choice for both acute and chronic AF was ointments application [26]. Furthermore, according to ASCRS guidelines, non-operative treatment of acute AFs should be the first-line approach [3]. Indeed, about half of the patients with acute AF has shown to respond to non-operative measures, which are well tolerated with minimal to no side effects [3]. In particular, topical CCBs, that have demonstrated a similar efficacy and a superior side-effect profile compared to topical nitrates [3], should be regarded as first-line treatment. Under this perspective, the administration of a topical combination including a CCB like NIF/LID should be considered a proper approach to the management of AF. NIF/LID is a treatment for AFs and proctalgia typically associated with anal sphincter hypertonia [20] which is available in Italy since 2004 [27]. Nifedipine, which belongs to CCBs class, inhibits the flow of calcium ions at the level of the smooth muscles of the anal sphincter, thus acting as a myorelaxant, by attenuating muscle contraction and internal anal sphincter hypertonia [28], as an anti-inflammatory, by suppressing the activation of the immune system [29], and as a microvascular regulator, by inhibiting the platelet-activating factor (PAF) [30]. Lidocaine, which is a local anesthetic, acts on pain by reversible blockade of nerve fiber impulse propagation stimulus at both the skin and deep levels: lignocaine binds to sodium channels, causing a conformational change that prevents the transient influx of sodium, therefore depolarization [31].The preliminary assessment revealed that NIF/LID was the most frequently prescribed treatment in relation with a diagnosis of AF. However, the distribution of study patients across the two subgroups showed a proportion of subjects prescribed with NIF/LID during the health encounter related to AF which is quite low (i.e., 14.0%). Besides this, patients receiving topical treatments specifically targeted for the treatment of AF (i.e., C05A drugs) during follow-up in the group of subjects without NIF/LID prescription at Index Date, despite being significantly higher than those found among patients with NIF/LID at Index Date, is lower than 7%. It is also worth mentioning that GTN accounted for less than 1.4% of treatments prescribed for AF, thus did not enter the top ten reported in Fig. 1S (Supplementary Materials); according to authors’ opinion, the low rate of GTN prescriptions observed might be attributable to GTN’s known side effects, and particularly headache [3, 8]. These findings, together with the even lower proportion of patients in this group receiving NIF/LID after Index Date (2.1%), might be suggestive of under-treatment of AFs in primary care. Important insights also came from the preliminary investigation on the ten molecules most frequently prescribed in relation with a diagnosis of AF, which showed mesalazine reaching the second position. Mesalazine is an anti-inflammatory drug used to treat inflammations of the digestive tract such as ulcerative colitis and mild-to-moderate Crohn’s disease, but it is not indicated to treat AF [21]. Similarly, beclomethasone, that ranked third, is indicated to treat colitis and ulcerative proctosigmoiditis [32]; diosmin combinations and hydrocortisone, which ranked, respectively, fourth and fifth, have the indication to treat hemorrhoids [33, 34]. The latter results might be suggestive of mis-prescription, which, in turn, might be related to misdiagnosis, which has been previously reported when dealing with common anal problems [35].

Results from the current analysis should be interpreted in the context of some limitations related to its retrospective and descriptive nature. First, a general limitation which is intrinsic of the data source used is one typical of real-world studies, that is, findings rely on the accuracy of recorded information, which is not ad-hoc collected for the study purposes, and is subject to potential for recording/coding errors or bias [36]. Indeed, identification of patients relied on proxies: the use of ICD-9 codes to identify AF cases might have introduced the potential for misclassification errors. However, GPs contributing to IQVIA LPD attend specific trainings for data entry and meet up-to-standard quality criteria related to the levels of coding. This, together with the high number of patients included in the study, makes authors confident that the extent of misclassification is mitigated and trends observed are real. Second, we cannot exclude that patients who were not prescribed with drugs of interest did not receive proper advice on hygienic and/or dietary conduct and/or on over-the-counter (OTC) medications. Unfortunately, information on hygienic and dietary regimen is not collected on the database, while that of OTC medications not requiring medical prescription for purchase is under-estimated on the database. Third, if a patient went to a specialist visit and the specialist prescribed a non-reimbursable drug to treat AF, it is possible that this did not result into a GP prescription, but the patient directly purchased the drug. Finally, IQVIA LPD database does not allow capturing secondary care interventions and surgical procedures such as botulin injections and LIS. However, considering the low counts of specialist visits referrals observed, authors believe that this limitation cannot have affected study’s results. This study also presents with important strengths, and the first one is represented by the use of real-world data coming from a very large database which has been already shown to be representative of the Italian general population [13]. Indeed, description of subjects with AF here reported is comparable with previous findings from scientific literature [10]. In addition, the adoption of GPs’ perspective allowed avoiding the selection bias that might affect studies conducted on people solely managed in the specialistic context that might, thus, offer a partial overview on AF. Scientific literature relying on real-world data to investigate management of AF is scarce [10]. Mapel and colleagues conducted a retrospective study on all persons enrolled in one large regional administrative database who received treatment for AF in the United Sates: surgical interventions were uncommon, while the majority of the patients were prescribed with topical treatments, even if it was found that many prescriptions were never filled [10]. AF management in Europe was investigated mainly through surveys which primarily involved the specialist setting. Balla and colleagues performed an international survey to investigate on practice and preferences of surgeons for AF treatment and found that the first treatment of choice for acute and chronic AF by colorectal surgeons was ointment application [26]. Aguilar and colleagues conducted a survey of surgeons of the Spanish Association of Coloproctology to evaluate the state of the art of the management of chronic AF in Spanish hospitals. Pharmacological treatments were found to be the first therapeutic step in 94% of the cases and among patients with hypertonia who were not at risk for fecal incontinence whereas 56% use hygienic-dietary measures together with GTN ointment [37]. Results from a national survey on French practices in the treatment of AF involving members of the French National Society of ColoProctology (SFNCP) showed that, despite members of the SNFCP agree with the importance of first-line medical treatment, CCBs and topical nitrates as first-step treatment were rarely prescribed in France, while priority was given to “simple” topical healing products and to on-demand oral analgesics [38]. A survey was conducted in the Netherlands to investigate the management of chronic AF and involved around 100 gastrointestinal surgeons and residents. Authors concluded that guideline recommendations are largely followed in the Netherlands: conservative measures, mainly fibers and/or laxatives and ointment, represented the first-line option, followed by surgical procedures [39]. In light of the pivotal role of medical treatments in the management of AF emerged from the previous investigations on doctors’ preferences, it is the authors opinion that the overview offered by the present study, which was based on real-world data reflecting actual prescription habits toward AF, is even more valuable. Finally, it is worth mentioning that findings here reported, and showing NIF/LID as the most frequently prescribed drug, fit with recommendations provided by international guidelines. Indeed, according to the Association of Coloproctology of Great Britain and Ireland, for the first-line treatment of chronic AF topical treatment for 2 months with either GTN 0.4% or diltiazem 2%, a CCB may be considered [7]. Moreover, according to the ASCRS, the use of CCBs for chronic AF has a similar efficacy, but a superior side effect, of topical nitrates, thus should be used as first-line treatment [3]. The variability observed when looking at the other treatments ranking among most frequently prescribed treatments is somewhat expected, as we are dealing with GPs data, and contributed underlining the need for a greater dialogue between GPs and specialists.

## Conclusion

The present study provides important insights on patients with anal fissure managed in primary care. Results showed that general practitioners are used to deal with this common anal pathology; however, there is still a gap between recommendations provided by guidelines and the actual management of anal fissure in primary care, which might represent room for improvements. Further studies involving primary data collection to better understand factors influencing general practitioner choice in terms of treatment option together with educational campaigns on the diagnosis of common anal problems might help further improving the management of this condition and optimizing health care resources utilization in primary care.

## Supplementary Information

Below is the link to the electronic supplementary material.

Supplementary file1 (DOCX 137 KB)

## Data Availability

The data that support the findings of this work are available from IQVIA but restrictions apply to the availability of these data, which were used under license for the current work, and so are not publicly available. Data are, however, available from the corresponding author upon reasonable request and with permission of IQVIA.
