# Supplementary material for: Description and management of patients with anal fissure: insights on Italian primary care setting coming from real-world data
Source: Updates Surg. 2024 May 26;76(6):2193–203. doi: 10.1007/s13304-024-01882-8 (PMC11541248; doi:10.1007/s13304-024-01882-8)
Supplement: Supplementary file 1 — Supplementary file1 (DOCX 137 KB) [file 13304_2024_1882_MOESM1_ESM.docx]

**Supplementary Materials**

**Updates in Surgery**

**Description and management of patients with anal fissure: insights on Italian primary care setting coming from real-world data**

Gaetano Gallo^1*^, Valeria Pegoraro^2^, Mario Trompetto^3^

^1^ Department of Surgery, Sapienza University of Rome, Rome, Italy

^2^ IQVIA Solutions Italy Srl, Milan, Italy

^3^ Department of Colorectal Surgery, S. Rita Clinic, Vercelli, Italy

Corresponding author^*^

Prof. Gaetano Gallo

Department of Surgery, Sapienza University of Rome, Rome, Italy

Address: Viale del Policlinico, 155, 00161 Roma RM

Tel: +39 328 438 5222

e-mail: [ga.gallo@uniroma1.it](mailto:ga.gallo@uniroma1.it)

ORCID-ID: orcid.org/0000-0003-1066-4671

***Fig1S*** *‘July 2015 – June 2022’ prescriptions for which the General Practitioner (GP) recorded a specific diagnosis of Anal Fissure (AF) stratified by molecule (TOP 10)*


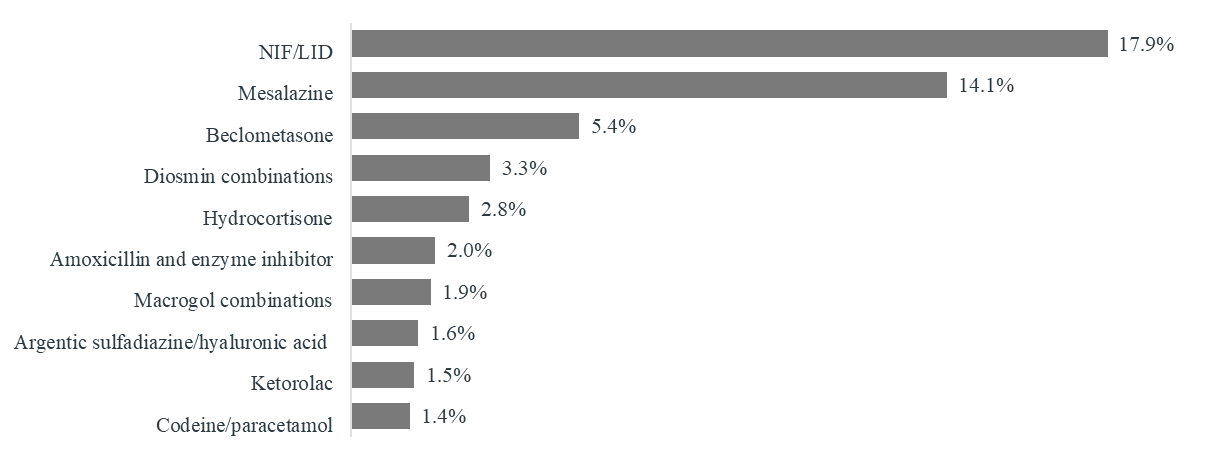


NIF/LID: nifedipine 0.3% in combination with lidocaine 1.5%.

*Table 1S: Diagnoses codes and examination referrals used to identify pregnancy status during baseline*

| **Diagnosis** | **ICD-9 Code** |
| --- | --- |
| *Normal pregnancy* | V22.xx |
| *Supervision of high-risk pregnancy* | V23.xx |
| *Ectopic and molar pregnancy* | 630.xx – 633.xx |
| *Other pregnancy with abortive outcome* | 634.xx – 639.xx |
| *Complications mainly related to pregnancy* | 640.xx – 649.xx |
| *Normal delivery and other indications for care in pregnancy, labor, and delivery* | 650.xx – 659.xx |
| *Complications occurring mainly in the course of labor and delivery* | 660.xx – 669.xx |
| *Complications of the puerperium* | 670.xx – 677.xx |
| *Other maternal and fetal complications* | 678.xx – 679.xx |
| **Referrals** | |
| *Obstetric ultrasound* | |
| *Obstetric visit* | |
| *Prenatal training* | |
| *Nuchal translucency* | |
| *Fetal echocardiogram* | |
| *Amniocentesis* | |
| *Fetal echocardiography* | |
| *Metaphase karyotype amniotic fluid* | |
| *Amniocytes culture* | |
| *Chorionic villi sample/culture* | |
| *Pregnancy-associated plasma protein (PAPP-A)* | |
| *Oral glucose challenge test during pregnancy (OGCT)* | |
| *Fetal flowmetry* | |
| *Amniotic fluid phospholipid chromatography* | |

ICD-9: International Classification of Diseases 9^th^ revision

*Table 2S: Diagnoses codes and examination referrals used to identify immunosuppressive condition during baseline*

| **Diagnosis** | **ICD-9 Code** |
| --- | --- |
| *Human immunodeficiency virus [HIV] disease* | 042.xx |
| *Organ or tissue replaced by transplant^a^* | V42.xx |
| *Neoplasms^b,c^* | 140.Xx – 239.xx |
| *Autoimmune Diseases^c,d^* |  |
| Rheumatoid arthritis and other inflammatory polyarthropathies | 714.xx |
| Psoriasis and similar disorders | 696.xx |
| Systemic lupus erythematosus | 710.0x |
| Systemic sclerosis | 710.1x |
| Sicca syndrome | 710.2x |
| Dermatomyositis | 710.3x |
| Multiple sclerosis | 340.xx |
| Pernicious anemia | 281.0x |
| Corticoadrenal insufficiency | 255.4x |
| Celiac disease | 579.0x |
| Toxic diffuse goiter | 242.0x |
| Chronic lymphocytic thyroiditis | 245.2x |
| Reiter’s disease | 099.3x |
| Type I Diabetes Mellitus | 250.01, 250.03, 250.11, 250.13, 250.21, 250.23, 250.31, 250.33, 250.41. 250.43, 250.51, 250.53, 250.61, 250.63, 250.71, 250.73, 250.81, 250.83, 250.91, 250.93 |
| Vitiligo | 709.01 |
| Regional enteritis | 555.xx |
| Ulcerative colitis | 556.xx |
| *Idiopathic fibrosing alveolitis^c^* | 516.3x |
| *Disorders involving the immune mechanism* | 279.xx |

ICD-9: International Classification of Diseases 9^th^ revision. ATC: anatomical therapeutic chemical classification.

*^a^* Patients with this condition were considered immunosuppressed only if at least one prescription falling into immunosuppressants class (ATC L04A) was also present during baseline

*^b^* Excluding 210.xx – 229.xx ‘Benign Neoplasms’

^c^ Patients with this condition were considered immunosuppressed only if at least one prescription falling in at least one among the following class was also present during baseline: immunosuppressants (L04A), corticosteroids for systemic use (H02)

^d^ At least one among the following codes

*Table 3S: Diagnoses codes used to identify comorbidities of interest during baseline*

| **Comorbidity** | **ICD-9 Code** |
| --- | --- |
| *Constipation* | 564.0x |
| *Diarrhea^a^* |  |
| Infectious Diarrhea | 009.2x |
| Diarrhea Of Presumed Infectious Origin | 009.3x |
| Functional Diarrhea | 564.5x |
| Diarrhea | 787.91 |
| *Diabetes mellitus* | 250.xx |
| *Essential hypertension* | 401.xx |
| *Hypothyroidism^a^* |  |
| Congenital hypothyroidism | 243.xx |
| Acquired hypothyroidism | 244.xx |
| *Obesity* |  |
| Overweight and obesity | 278.0x |
| *Anxiety* | 300.0x |
| *Depression^a^* |  |
| Major depressive disorder, single episode | 296.2x |
| Major depressive disorder, recurrent episode | 296.3x |
| Depressive disorder, not elsewhere identified | 311.xx |
| *Chronic inflammatory bowel diseases^a^* |  |
| Regional enteritis | 555.xx |
| Ulcerative colitis | 556.xx |
| *Heart diseases^a^* |  |
| Chronic rheumatic heart disease | 393.xx – 398.xx |
| Hypertensive heart disease | 402.xx |
| Hypertensive heart and chronic kidney disease | 404.xx |
| Ischemic heart disease | 410.xx – 414.xx |
| Acute pulmonary heart disease | 415.xx |
| Chronic pulmonary heart disease | 416.xx |
| Other forms of heart disease | 420.xx – 429.xx |

ICD-9: International Classification of Diseases 9^th^ revision. BMI: body mass index.

*^a^* At least one among the following codes.

*^b^* Also presence of a registration of BMI≥30 kg/m^2^ during baseline contributed to the definition of obesity

***Fig2S*** *Details on follow-up drugs prescriptions and diagnostic examinations. Analysis on the overall cohort and on sub-cohorts defined by presence/absence of nifedipine 0.3% in combination with lidocaine 1.5% (NIF/LID) prescriptions at Index Date*


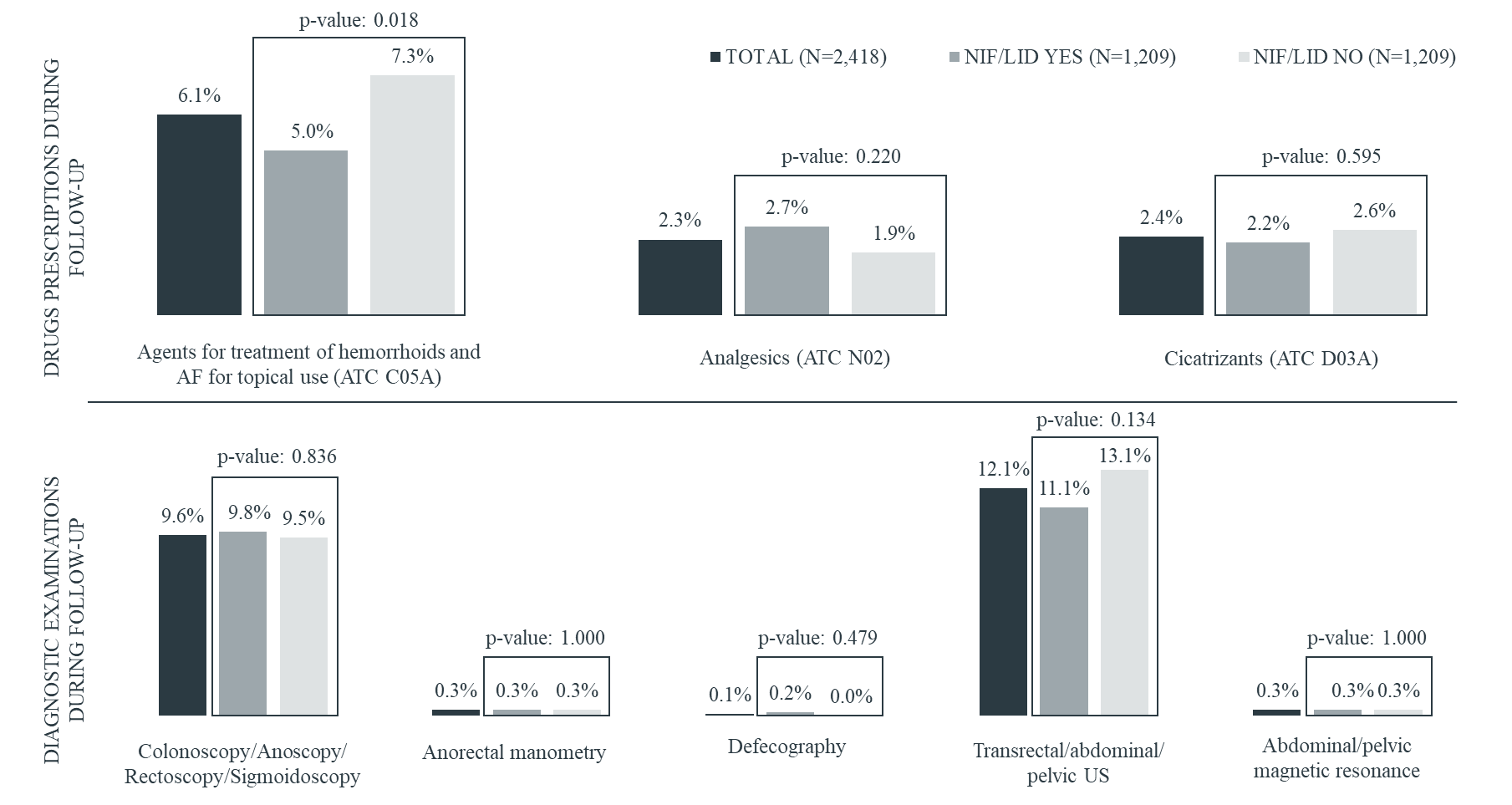


NIF/LID: nifedipine 0.3% in combination with lidocaine 1.5%. ATC: anatomical therapeutic chemical classification.
